# NOVEL APPROACHES FOR CROSS-NATIONAL COGNITIVE AGING RESEARCH

**DOI:** 10.1093/geroni/igae098.2078

**Published:** 2024-12-31

**Authors:** Alden Gross, Lindsay Kobayashi, Kenneth Langa

**Affiliations:** Chair; Co-Chair; Discussant

## Abstract

Cross-national comparisons of cognitive outcomes are increasingly feasible given availability of newer data resources. Given a broad, global study population, cross-national research may uncover mechanistic insights that would not be clear from one country or a socioeconomically homogeneous set of countries. Despite its promise, cross-national data present with numerous methodological and interpretational challenges, which will be highlighted in this symposium. In this symposium, Kobayashi and Gross (co-chairs) will lead with best practices for cross-national research, highlighting findings from the Harmonized Cognitive Assessment Protocols (HCAPs), a major NIA-funded platform for valid cross-national comparisons of ADRD. Cai will present a novel platform for deriving classification algorithms for dementia, based on neuropsychological criteria standardized to a robust normative group. Leveraging HCAP studies from the US, England, and India, they applied variations of classification algorithms. Next, Wu and colleagues, using longitudinal data from China’s HCAP (N=6,830), present a challenge–form differences introduced by changing test stimuli across waves–and a solution–weighted equipercentile equating–for when parallel but non-equivalent memory tests are administered longitudinally in surveys. Third, out of recognition that culturally diverse and low-resource settings can affect reliability and validity of cognitive tests, Mani and colleagues present psychometric properties of a brief pilot HCAP in Kenya (N=203). Finally, Zhang will present simulation results and demonstration of an R/Shiny-App regarding effects of differential pre-baseline selection on inferences for cross-national studies. Pre-baseline selection refers to selective survival in a target population occurring before a study’s baseline, and can vary dramatically between high-income and low-income countries.

